# Respiratory viruses in pediatric emergency department patients and their family members

**DOI:** 10.1111/irv.12789

**Published:** 2020-07-30

**Authors:** Nelsa Matienzo, Mariam M. Youssef, Devon Comito, Benjamin Lane, Chanel Ligon, Haruka Morita, Arianna Winchester, Mary E. Decker, Peter Dayan, Bo Shopsin, Jeffrey Shaman

**Affiliations:** ^1^ Environmental Health Sciences Department Mailman School of Public Health Columbia University New York NY USA; ^2^ Division of Infectious Diseases Department of Medicine NYU School of Medicine New York NY USA; ^3^ Department of Pediatrics Columbia University Irving Medical center New York NY USA

**Keywords:** influenza‐like illness, respiratory viral infection, seasonal pattern

## Abstract

**Background:**

Respiratory viral infections account for a substantial fraction of pediatric emergency department (ED) visits. We examined the epidemiological patterns of seven common respiratory viruses in children presenting to EDs with influenza‐like illness (ILI). Additionally, we examined the co‐occurrence of viral infections in the accompanying adults and risk factors associated with the acquisition of these viruses.

**Methods:**

Nasopharyngeal swab were collected from children seeking medical care for ILI and their accompanying adults (Total N = 1315). Study sites included New York Presbyterian, Bellevue, and Tisch hospitals in New York City. PCR using a respiratory viral panel was conducted, and data on symptoms and medical history were collected.

**Results:**

Respiratory viruses were detected in 399 children (62.25%) and 118 (17.5%) accompanying adults. The most frequent pathogen detected was human rhinovirus (HRV) (28.81%). Co‐infection rates were 14.79% in children and 8.47% in adults. Respiratory syncytial virus (RSV) and parainfluenza infections occurred more often in younger children. Influenza and HRV occurred more often in older children. Influenza and coronavirus were mostly isolated in winter and spring, RSV in fall and winter and HRV in fall and spring. Children with HRV were more likely to have history of asthma. Adults with the same virus as their child often accompanied ≤ 2‐year‐old‐positive children and were more likely to be symptomatic compared to adults with different viruses.

**Conclusions:**

Respiratory viruses, while presenting the same suite of symptoms, possess distinct seasonal cycles and affect individuals differently based on a number of identifiable factors, including age and history of asthma.

## 1 INTRODUCTION

Respiratory infections account for a substantial fraction of emergency department (ED) visits,^1^ particularly among the pediatric population.^2^ Respiratory diseases were among the most common reasons for pediatric ED visits in the US during 2015, of which acute upper respiratory tract infections (RTI) were the most common.^3^ Despite major public health efforts, epidemics of viral RTI continue to be highly prevalent among healthy populations with potential lethal consequences in susceptible individuals.^4^ Advances in laboratory testing for RTI have become widely available.^5^ However, clinical decision regarding RTI is usually based on presumptive diagnosis, which can be challenging due to the similarity of symptoms exhibited by different respiratory viruses.^6^ Identifying epidemiological characteristics of respiratory viral infections may therefore help clinicians better manage their patients.^7^

Infection rates for different respiratory viruses vary across age groups in children.^8^ Although there is consistency in the literature about higher risk of respiratory syncytial virus (RSV)^9^ and parainfluenza virus (PIV)^10^ in infants, findings are mixed regarding other respiratory viruses. For example, McDermott, et al^3^ found that influenza is more prevalent in children less than one‐year‐old, whereas Taylor, et al^11^ reported that influenza infection rates increase in children older than 5 years. More research is thus needed to capture the distribution of common respiratory viruses across different age groups. Ultimately, this may help advance targeted control and prevention of RTI.^12^

Most respiratory viruses possess distinct seasonal cycles.^13^, ^14^ For example, influenza and coronavirus are known to be prominent in the winter.^13^, ^14^, ^15^ Peak prevalence for HRV has been observed in the spring and fall.^16^ Identification of the seasonality of common respiratory viruses may aid in determining appropriate precautions that can curb the morbidity and mortality rates during peak transmission months.^17^

Children often carry respiratory viruses to their homes and spread infection to their families. Alternatively, family members occasionally expose their child to viruses.^18^ Influenza‐like illness has been reported in 12% of the adult family members of the children with a laboratory‐detected viral pathogen and viral pathogens have been detected in 42.3% of symptomatic family members.^19^

In the current study, we analyzed swab specimens from children seeking medical care in pediatric EDs for acute respiratory illness, as well as from adults who accompanied these children and were living in the same household. We examined the prevalence trends of seven common respiratory viruses, which often produce a suite of non‐specific respiratory symptoms—commonly referred to as influenza‐like illness (ILI), including influenza, coronavirus, human metapneumovirus (hMPV), HRV, PIV, RSV, and adenovirus. Additionally, we examined the age and seasonal distribution of these respiratory viral infections in children, the co‐occurrence of viral infections in accompanying adults, and potential risk factors for viral RTIs.

## 2 METHODS

### 2.1 Sample collection and clinical data

Participants were recruited at three Pediatric EDs, New York Presbyterian (NYP), Bellevue, and Tisch hospitals in New York, NY, from August 2016 to May 2018. Nasopharyngeal swabs were collected from children and teenagers aged 0‐19 years old who were brought to the ED with an ILI—defined as a fever of 100°F or greater and a cough or sore throat in the absence of other diagnosis.^20^

Additionally, adults who accompanied children to the ED visit and were living in the same household were recruited and swabbed, regardless of symptoms. A detailed epidemiological questionnaire was obtained that included information on demographics, clinical presentation, medical history, immunizations (including influenza vaccination history for the previous 5 years), and living situation. Parents provided informed consent and completed the survey for children under 13. The study protocol was approved by Institutional Review Board of Columbia University Medical Center (Protocol AAAQ4358). Participants were asked whether they had experienced symptoms commonly associated with respiratory virus infection during the 48 hours prior to completing the survey, and to rate those symptoms as mild, moderate, or severe. These symptoms included fever, chills, muscle paints, watery eyes, runny nose, sneezing, sore throat, cough, and chest pain.

One nasopharyngeal swab was collected from each participant using a minitip flock swab (VWR catalog no. 10755‐196; Copan Diagnostics) and immediately placed in a 15 mL tube containing 2 mL of DNA/RNA Shield (Product no. R1100‐250; Zymo Research) to inactivate any infectious agents while preserving the genetic integrity of the sample.

### 2.2 Virus detection and identification

Samples were stored at −4°C for no more than 30 days, then separated into two 1 mL aliquots, placed in 1.5 mL cryogenic vials, and stored at −80°C. Samples were processed according to the GenMark Dx eSensor® Respiratory Viral Panel (RVP) Package Insert (bioMerieux). Automated nucleic acid extraction was performed using bioMérieux NucliSENS^®^ easyMAG^®^. Viruses were detected using eSensor XT 8 Respiratory Viral Panel (RVP; GenMark Diagnostics), a multiplex PCR assay. The GenMark eSensor XT‐8 detects the following virus types and subtypes: Influenza A, Influenza A H1 Seasonal Subtype, Influenza A H3 Seasonal Subtype, Influenza A 2009 H1N1 subtype, Influenza B, Coronavirus, subtypes 229E, NL63, OC43, and HKU1, RSV A, RSV B, PIV 1, PIV 2, PIV 3, hMPV, HRV, Adenovirus B/E, and Adenovirus C.

### 2.3 Statistical analysis

Data were statistically evaluated using IBM SPSS Statistics (SPSS Inc, Version 26.0). Analyses focused only on viral types and were not broken down by subtype due to the low numbers for many subtypes. Comparison of demographic and clinical characteristics was performed using two‐tailed *t*‐tests for continuous variables and chi‐square analyses for categorical variables. For the seasonality analysis, samples collected from December to February were designated as winter, March to May as spring, June to August as summer, and September to November as fall. For the age‐wise distribution analysis, children were divided into four age groups: <2 years old, 2‐4 years old, 5‐12 years old, and 13‐19 years old.

## 3 RESULTS

### 3.1 **Sample characteristic**s

A total of 1315 samples were collected from the three Pediatric EDs. These included 641 children and 674 accompanying family members. Table 1 shows detailed characteristics of the study participants.

**Table 1:** Characteristics of study sample

	Children (N = 641)		Adults (N = 674)	
	Mean	SD	Mean	SD
Age	5.82	4.80	33.05	10.62
Number of household members	3.05	1.7	2.94	1.57
	**N**	**%**	**N**	**%**
Gender				
Male	322	50.23	127	18.8
Female	316	49.30	539	80
Transgender	2	0.31	6	0.9
Not known	1	0.16	2	0.30
Ethnicity:				
Hispanic	524	81.75	464	68.84
Not Hispanic	117	18.25	210	31.16
Smoking				
Current smoker	0/599	0	64/673	9.51
History of smoking in household	33/528	6.25	41/650	6.31
Presence of pets	172	26.83	192	28.49
History of influenza vaccine in the same or preceding year	388/598	64.88	368/672	54.76
History of asthma	231/598	38.63	109/672	16.22
History of any allergic diseases	140/598	23.41	159/672	23.66
Laboratory positive for one or more viral infections	399	62.25	118	17.51

### 3.2 Viral prevalence

From the whole sample, 39.32% (N = 517; 399 children and 118 accompanying adults) tested positive for one or more of the seven viruses. Among positive samples, HRV was detected in 28.81%% of total infections, PIV in 17.12%, RSV in 15.42%, influenza in 15.25%, adenovirus in 9.49%, hMPV in 5.93%, and coronavirus in 7.97%. The co‐infection rate was 14.79% (N = 59) in positive children and 8.47% (N = 10) in positive adults. HRV was the most frequently detected co‐infecting virus (23.7%), followed by adenovirus (19.26%). The least frequent co‐infecting virus was hMPV (5.19%).

### 3.3 Characteristics of positively tested children

Three hundred and ninety nine positive samples were collected from children in the three pediatric EDs. Out of these, 50.12% were males, 49.6% were females, and one child was transgender. No significant gender differences were observed among all respiratory viruses.

**FIGURE 1:**
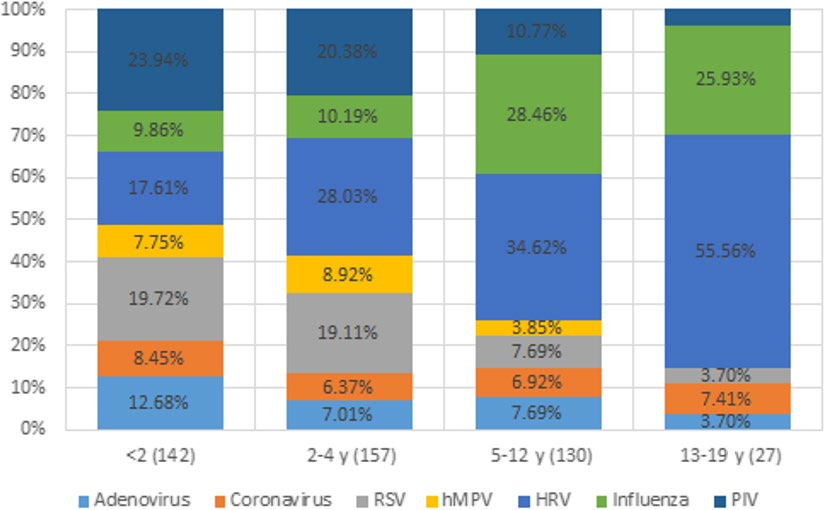
Prevalence of different respiratory viruses among children with acute respiratory infections in different age groups

Children were divided into four age groups: <2 years old (143 infections), 2‐4 years old (162 infections), 5‐12 years old (130 infections), and 13‐19 years old (27 infections). RSV and PIV infections occurred significantly more often in younger children; (χ^2^ = 16.01, *P* < .001) and (χ^2^ = 16.16, *P* < .001), respectively. Influenza and HRV infections occurred significantly more often in older children; (χ^2^ = 17.94, *P* < .001) and (χ^2^ = 16.01, *P* < .001), respectively. Significant differences across age groups were not observed for other viral infections (Figure 1). Because the 13‐19‐year‐old age group included only 27 children, the above analyses were repeated excluding this age group and yielded similar findings.

Among children testing positive for a single viral infection, influenza or PIV infections were significantly associated with fever; (χ^2^ = 14.44, *P* = .00014) and (χ^2^ = 8.15, *P* = .003), respectively. Further, children infected with HRV were less likely to have fever compared with children with other viruses (χ^2^ = 5.52, *P* = .02).

### 3.4 Differences among infections in children with and without asthma

**FIGURE 2:**
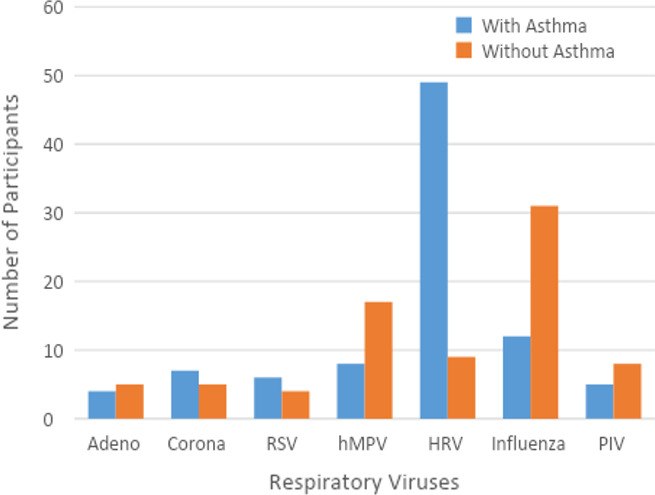
Distribution of respiratory viruses in asthmatic and non‐asthmatic children aged ≥ 5 years

Given the difficulty in making a definitive diagnosis of asthma in children under five years of age,^21^ we only included children over five (N = 307) in this analysis. Of these children, there were 151 who had been previously diagnosed with asthma, and 141 children who tested positives for one or more respiratory viruses, of whom 71 had history of asthma (Figure 2).

There was no difference in rate of viral infection between asthmatic and non‐asthmatic children (χ^2^ = 1.75, *P* = .2). HRV infections occurred more frequently in children with asthma (χ^2^ = 29.54, *P* = 5.46E‐07). However, influenza infections occurred more frequently in children without asthma (χ^2^ = 12.41, *P* = .000427). Because children with a history of asthma are more likely to get an influenza vaccine,^22^ we examined whether receiving an influenza vaccine in the same or the preceding year was different between the asthmatic and non‐asthmatic children. Interestingly, we found that asthmatic children were more likely to have received their influenza vaccination, compared with non‐asthmatic children (χ^2^ = 7.15, *P* = .009). There were no significant asthma‐related differences observed for the other viruses.

### 3.5 Seasonality of respiratory viruses in pediatric patients and their family members

Sampling was performed year‐round during 2016 and 2017 only at NYP Pediatric ED (N = 994). Of these samples, 472 (47.48%) tested positive for one or more viruses whereas 522 (52.52%) tested negative. Of the positive samples, 403 (85.38%) tested positive for one virus, 66 (13.98%) tested positive for two viruses, and 3 samples (0.63%) tested positive for three viruses.

**FIGURE 3:**
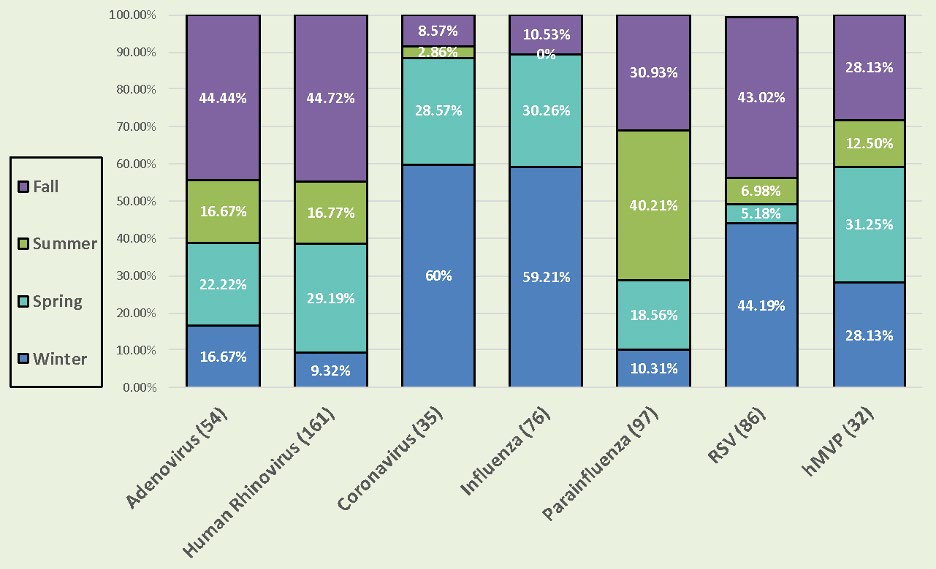
Seasonality of respiratory viruses in nyp pediatric emergency department patients and their family members

As shown in Figure 3, the majority of coronavirus (88.57%) and influenza (89.47%) infections were observed during winter and spring. Most HRV infections (73.91%) occurred during fall and spring. Most PIV infections (71.14%) occurred during summer and fall, and most RSV infections (88.21%) occurred during fall and winter. hMPV infections were observed near equivalently during fall, winter, and spring. Approximately, 44% of adenovirus infections occurred during fall and the remainder were observed at similar rates during other seasons. Collectively, with the exception of hMPV and adenovirus, the majority of respiratory viral infections detected in the current study occurred during only two 3‐month seasons/year.

### 3.6 Symptomology of accompanying adults

Among 674 accompanying adults in the 3 pediatric EDs, 118 (17.50%) were positive for at least one respiratory virus. Accompanying adults were subdivided into two groups: those with the same virus type (and subtype) as their child (N = 72, 10.68%), and those with a different virus or who tested positive while their child tested negative (N = 46, 6.82%).

Of the 72 accompanying adults who tested positive for the same virus as their child, 46 were symptomatic (63.88%). Of the 46 accompanying adults who had a different virus or a negative child, 20 were symptomatic (43.48%). Adults who tested positive for the same virus as their child were significantly more likely to have experienced ILI symptoms in the preceding 48 hours, compared with adults who either had a different virus than their child or a child that tested negative (χ2 = 4.7439, *P* = .029402).

Among all viruses, we found that adenovirus was detected in 30% of the adults accompanying their adenovirus‐positive children. However, other viruses were only detected in less than 20% of the accompanying adults of relevant positive children.

Age of accompanying adults and history of medical comorbidities were not significantly related to occurrence of viral infection. History of asthma was not associated with infection by any respiratory virus—although this finding may be a result of the small number of adults infected with each virus separately. More accompanying adults with a history of asthma received an influenza vaccine in the same or preceding year than non‐asthmatic accompanying adults (χ^2^ = 8.03, *P* = .006). Adults who accompanied < 2‐year‐old‐positive children were more likely to have same virus as their child (27.7%) compared with adults who accompanied ≥ 2‐year‐old‐positive children (13.9%) (χ2 = 10.7576, *P* = .00104).

Families with a household number of ≥ 5 were more likely to have a child and/or accompanying adult test positive for one or more respiratory viruses, compared to families with households of < 5 (χ^2^ = 4.08, *P* = .04). There was no effect of smoking or presence of pets in relation to either acquiring infection in children and/or in adults.

## 4 DISCUSSION

Respiratory viral infections are one of the leading causes of pediatric ED visits.^23^, ^24^ Understanding their underlying epidemiology is crucial for promoting preparedness to tackle this public health problem.^25^ In the current study, we report on the viral etiology that commonly affect children seeking ED with ILI symptoms, as well as addressing their epidemiological features.

We detected at least one of the tested respiratory viruses in two‐thirds of children and 18% of their accompanying adults enrolled in the current study. The most frequent pathogen detected was HRV, which is consistent with previous studies in the United States^26^ and other countries.^11^

Among the virus‐positive cases in our study, the co‐infection rate was around 15% in children. Previous studies are mixed regarding co‐infection rates of respiratory viruses in children. While some studies have reported similar figures to ours,^27^ higher^28^, ^29^, ^30^ and lower^31^ rates have also been reported, probably due to different methodological approaches and geographical distribution among these studies. HRV and PIV were detected in co‐infected patients more frequently than other viruses, probably because the incidence of both viruses was higher than that of the other respiratory viruses. Further, infections from both of these viruses were abundant during autumn, which may also have contributed to this finding. Among all co‐infecting viruses, HRV was the most prevalent virus, consistent with some prior studies.^30^, ^32^

We found that RSV and PIV infection rates are significantly higher in younger children, a trend that has been seen in other studies.^10^, ^33^, ^34^, ^35^ RSV and PIV infect cells in the epithelium lining of the trachea and intrapulmonary airways, and cause croup, bronchitis, bronchiolitis, and/or bronchopneumonia.^36^ Young infants have small airways, which make them susceptible to obstruction, and, in turn, increase risk for RSV and PIV infection specifically.^37^, ^38^

Additionally, we found that HRV and influenza infection rates increased significantly as children get older. Past influenza epidemiological studies have mixed findings with some showing younger children experiencing higher infection rates, whereas others reporting higher rates in older children.^3^, ^39^, ^40^, ^41^ Here, influenza infections appear to be much more common in school‐aged children, ages 5‐19, which may be related to elevated contact rates during the school year. Regarding HRV, this study does not agree with past studies, which found higher rates of HRV infection in younger children.^42^, ^43^ However, those studies only looked at children 0‐5 years old, whereas the current study examined children up to 19 years old. It should be noted also that our findings only account for infections that warranted an ED visit, and thus do not take into account asymptomatic children or those with mild infections.

Precautionary and therapeutic measures for respiratory viruses are usually taken based largely on their seasonality patterns.^44^ Consistent with previous studies,^13^, ^14^, ^45^, ^46^, ^47^, ^48^ we found that more than 88% of coronavirus and influenza infections occurred during winter and spring. Further, around 75% of HRV infections were observed during fall and spring, probably indicating bi‐annual peaks of HRV in relation to return to school—and onset of increased contact among children—in the fall and spring.^49^, ^50^ While most previous studies reported that PIV is prevalent in fall, summer, and spring—with some variations in relation to PIV serotypes—we found that most PIV infections occurred during summer and fall. However, the current study lumps together PIV serotypes. Lastly, as previously documented,^10^, ^51^, ^52^, ^53^ adenovirus had no seasonal pattern in the current study.

Regarding the relationship between history of asthma and respiratory viral infection, we observed significant relationships between both HRV and influenza infection rates and history of asthma. Specifically, most patients seeking medical care for HRV were asthmatic. HRV promotes the clinical features of asthma, including airway obstruction, airway inflammation, and airway hyper responsiveness ,^54^ which may explain our finding. On the other hand, influenza was more frequent in non‐asthmatic children, possibly contradicting previous studies showing that asthmatic children were at greater risk of complications due to influenza.^40^, ^43^ A possible explanation for our finding is that children with history of asthma are more likely than others to get an influenza vaccine,^22^, ^55^ which is the case in the current study as well.

Interestingly, 118 of the adults who accompanied their children tested positive for at least one of the respiratory viruses examined in the current study. Approximately 60% of these positive accompanying adults had the same virus as their children, the majority of whom were < 2‐year‐old‐positive children. Consistent with this finding, our group has previously shown that adults who are in daily contact with children have a higher number of infections compared to their counterparts without daily contact with children.^13^ Households with same virus as their index child were more likely to have symptomatic infections compared with households with a different virus or a negative index child. This may indicate a relationship between symptoms expression and contagion of the virus.^56^ Of note, around one third of the accompanying adults who tested positive with same virus as their symptomatic children did not report symptoms of RTI. A possible explanation of this finding is that appearance of symptoms depends not only on the viral cause but also on host response.

We found a high rate of adenovirus infection in families with an adenovirus‐positive child. This is consistent with one prior study that showed a high secondary attack rate for adenovirus.^57^ However, our sample size of adenovirus‐positive cases was modest and studies with larger sample size are needed to draw a definitive conclusion.

### 4.1 Limitations

The current study has some limitations. First, due to the low number of viral subtypes in the current sample, we conducted analyses only on viral types without breakdown by subtype. Additionally, we studied the rate of viral infections in adults who accompanied children seeking care for ILI symptoms at the time of ED visit. This did not allow for calculation of the secondary attack rate per index child. We also did not have data on whether the children were attending childcare centers, which represent a common source of infection. Further, for this study, we only recruited children with ILI symptoms, a subset of children visiting the EDs; consequently, whether the study findings are representative of the full population cannot be concluded because the total number of patients during the study period is not available. Finally, clinical outcomes for the participants were not available to evaluate the prognosis of the respiratory viruses among children with ILI attending EDs in New York.

## 5 CONCLUSIONS

The epidemiological data on common circulating respiratory viruses in this study could provide helpful information for clinical decision‐making. Findings can inform guidelines for clinicians by enabling reasonable estimates of etiologic diagnoses and identification of individuals who are more susceptible to a particular virus and when they are at greatest risk.

## CONFLICT OF INTEREST

Dr. Shaman reports Personal fees from Business Network International, other from SK Analytics, outside the submitted work. Dr. Shopsin reports Personal fees from MicroGenDx, outside the submitted work. Other authors have no conflict of Interest to declare.

## AUTHOR CONTRIBUTION

**Nelsa Matienzo:** Conceptualization (equal); Data curation (equal); Formal analysis (equal); Methodology (equal); Visualization (equal); Writing‐original draft (equal). **Mariam M. Youssef:** Conceptualization (equal); Data curation (equal); Formal analysis (equal); Software (equal); Validation (equal); Visualization (equal); Writing‐original draft (equal); Writing‐review & editing (equal). **Devon Comito:** Methodology (supporting). **Benjamin Lane:** Methodology (supporting); Project administration (supporting). **Chanel Ligon:** Methodology (supporting); Project administration (supporting). **Haruka Morita:** Methodology (supporting); Project administration (supporting). **Arianna Winchester:** Methodology (supporting); Project administration (supporting). **Mary Decker:** Methodology (supporting); Project administration (supporting). **Peter Dayan:** Methodology (supporting). **Bo Shopsin:** Conceptualization (supporting); Writing‐review & editing (lead). **Jeffrey Shaman:** Conceptualization (lead); Funding acquisition (lead); Investigation (lead); Methodology (lead); Resources (lead); Supervision (lead); Writing‐review & editing (lead).
